# The Evolution of the Antimicrobial Resistance of *Streptococcus pneumoniae* in Tunisia: A Multicentric Analysis over Two Decades (2000–2019)

**DOI:** 10.3390/antibiotics14020171

**Published:** 2025-02-10

**Authors:** Nourelhouda Ben Ayed, Omar Gargouri, Samar Mhimdi, Fahmi Smaoui, Emna Mhiri, Lamia Kanzari, Meriam Zribi, Senda Maalej Mezghanni, Sonia Ktari, Khaoula Meftah, Naglaa Mohamed, Hela Zaghden, Olfa Bahri, Sophie Besbes, Wafa Achour, Leila Slim, Ilhem Boutiba, Hanen Smaoui, Adnene Hammami

**Affiliations:** 1 Laboratory of Microbiology, Habib Bourguiba University Hospital Center, Sfax 3000, Tunisia; bayednour@yahoo.fr (N.B.A.); omar.gargouri@medecinesfax.org (O.G.); maalejsenda69@gmail.com (S.M.M.); 2 Research Laboratory LR03SP03 “Micro-Organisme et Pathologie Humaine”, Faculty of Medicine, University of Sfax, Sfax 3029, Tunisia; smaouifahmi@yahoo.fr (F.S.); sonia_ktari2002@yahoo.fr (S.K.); 3 Laboratory of Microbiology, Bechir Hamza Children’s Hospital, Tunis 1006, Tunisia; samarmem@gmail.com (S.M.); meftahkhaoula@gmail.com (K.M.); hanen.smaoui@gmail.com (H.S.); 4 Laboratory of Microbiology, Abderrahmen Mami Hospital, Ariana 2080, Tunisia; emna.mehiri@tunet.tn (E.M.); leilaslimsaidi@gmail.com (L.S.); 5 National Reference Laboratory on Antimicrobial Resistance Surveillance, Tunis 1007, Tunisia; lamiakanzari@yahoo.fr (L.K.); ilhem.boutiba@gmail.com (I.B.); 6 Microbiology Laboratory, Charles Nicolle Hospital, Tunis 1006, Tunisia; 7 Research Laboratory “Antimicrobial Resistance” LR99ES09, Faculty of Medicine of Tunis, University of Tunis El Manar, Tunis 1007, Tunisia; 8 Laboratory of Microbiology, La Rabta Hospital, Tunis 1007, Tunisia; meriamzribimiled@gmail.com; 9 Pfizer Inc., New York, NY 10001-2192, USA; naglaa.mohamed@pfizer.com; 10 Pfizer Inc., Tunis 1053, Tunisia; hela.zaghden@pfizer.com; 11 Laboratory of Clinical Biology, Aziza Othmana Hospital, Tunis 1008, Tunisia; olfa.bahri@fmt.utm.tn; 12 Laboratory of Microbiology, Mohamed Kassab Orthopaedics Institute, Manouba 2010, Tunisia; sophia.besbes@yahoo.fr; 13 Laboratory Department, Bone and Marrow Transplantation Center, Tunis 1029, Tunisia; wafa.achour@gmail.com

**Keywords:** *Streptococcus pneumoniae*, antimicrobial resistance, Tunisia

## Abstract

**Background/Objectives**: *Streptococcus pneumoniae* is a leading respiratory pathogen responsible for significant morbidity and mortality, particularly among vulnerable populations. Understanding its antimicrobial resistance patterns and serotype distribution is crucial for guiding treatment and prevention strategies. This study aims to examine these trends in *S. pneumoniae* isolates from Tunisia over a two-decade period (2000–2019). **Methods**: A retrospective time series analysis was conducted on data (n = 4284) gathered from eight university hospital centers across Tunisia. Antimicrobial susceptibility testing was performed according to the European Committee on Antimicrobial Susceptibility Testing (EUCAST) guidelines. Pneumococcal serotypes were determined for a subset of samples from 2012 to 2019 (n = 903) using multiplex PCR and latex agglutination. **Results**: Penicillin G resistance decreased from 9–13.7% during 2000–2002 to 4.3% by 2019, while amoxicillin resistance increased until reaching 10% in 2019. Erythromycin resistance initially increased before stabilizing between 61.9% and 66.3% during 2014–2019, whereas tetracycline resistance declined from 2000 to 2008 and fluctuated around 40% during 2009–2019. Levofloxacin resistance did not exceed 1.2% throughout the study period. The most prevalent serotypes were 14, 19F, 19A, 23F, 3, 6B, 6A, and 9V. Among them, serotype 3 was the most susceptible overall. Serotypes 23F, 14, 9V, and 6B displayed the highest levels of multi-drug resistance. **Conclusions**: Penicillin G (high-dosage), cefotaxime, and levofloxacin are still effective against most *S. pneumoniae* strains in Tunisia, while erythromycin and tetracycline are not reliable options for treating pneumococcal infections. Alarming resistance rates among prevalent serotypes, except serotype 3, underscore the need for preventive measures, rational antibiotic use, and ongoing surveillance.

## 1. Introduction

*Streptococcus pneumoniae* is a prevalent pathogen in the respiratory tract that can lead to significant morbidity and mortality. This bacterium causes a wide range of infections, from non-invasive diseases like otitis media and sinusitis to invasive pneumococcal diseases (IPDs) such as bacteremia and meningitis [1,2,3]. Although more than 100 serotypes of *S. pneumoniae* have been identified based on its polysaccharide capsule, few of them are predominantly implicated in pneumococcal diseases [1,4]. The introduction of pneumococcal conjugate vaccines (PCVs) such as PCV-7 and PCV-13 has effectively reduced the prevalence of pneumococcal diseases caused by vaccine serotypes in children, as well as in adults [5,6].

The rise in antimicrobial resistance (AMR) among *S. pneumoniae* is a major therapeutic challenge for treating pneumococcal diseases [2,7]. Globally, there has been a significant increase in the prevalence of *S. pneumoniae* isolates resistant to ß-lactams (penicillin and cephalosporins) and macrolides [6,8]. Following the implementation of PCVs, a decline in the rate of AMR among pneumococcal strains has been observed globally [7,9]. PCVs lower AMR directly by preventing pneumococcal infections and indirectly by reducing antibiotic consumption, thereby mitigating the selective pressure on resistant strains [9]. Meanwhile, the emergence of resistance in non-vaccine serotypes has become a concern [7,9]. Moreover, increased antibiotic consumption among individuals with chronic infections could exacerbate antibiotic resistance [3]. Community-acquired pneumonia (CAP) treatment contributes to 30% to 50% of all antibiotic utilization [10]. In 2019 alone, bacterial AMR was associated with 4.95 million deaths, with 1.27 million deaths directly linked to this challenge [5]. *S. pneumoniae* ranks among the top six pathogens responsible for the highest rates of global mortality associated with AMR [5].

*S. pneumoniae* is a commonly identified pathogen in cases of CAP and bacterial meningitis in Tunisia [11]. It was projected that pneumococcal pneumonia and pneumococcal meningitis lead to 1091 and 69 hospitalizations annually, respectively, resulting in a total expenditure of around USD 256,000 [11]. A high level of multi-drug resistance has been observed among *S. pneumoniae* isolates in Tunisia. The non-susceptibility rates towards penicillin, amoxicillin, cefotaxime, and erythromycin have been documented at 67.9%, 45.3%, 16%, and 62.3%, respectively [12]. Another study reported that 54.8% of *S. pneumoniae* isolates were multi-drug resistant (MDR). Among these MDR strains, the highest non-susceptibility rates were observed for β-lactams (98.6%), macrolides (99.6%), and tetracycline (75.4%) [13]. Continuous monitoring of resistance trends in *S. pneumoniae* is essential for effective infection treatment and for developing public health strategies to combat antibiotic resistance. However, there is a lack of large national surveillance studies that assess the evolutionary trends of *S. pneumoniae* resistance in Tunisia. The Antimicrobial Resistance Surveillance Program (LART) has tracked the trends of antimicrobial resistance throughout Tunisia since 1999.

This study aims to describe the evolution of *S. pneumoniae* resistance, phenotypic profiles, and serotype distribution over two decades, spanning from 2000 to 2019, in Tunisia.

## 2. Results

### 2.1. Characteristics of S. pneumoniae Isolates

A total of 4284 pneumococcal isolates were collected from 8 participating centers between 2000 and 2019 in Tunisia. Of these isolates, 1247 (29.1%) were invasive and 3037 (70.9%) were non-invasive isolates. Approximately 1802 isolates (42.1%) were recovered from children and 2000 (46.7%) from adults, while the age of patients was unknown for 468 isolates. Most isolates were cultured from the lower respiratory tract (n = 2262, 52.9%), while the other isolates were mainly obtained from blood culture (n = 449, 10.5%), CSF (n = 423, 9.9%), and the upper respiratory tract (n = 270, 6.3%).

### 2.2. Overall Evolution of S. pneumoniae Antimicrobial Resistance

#### 2.2.1. Resistance Trends to β-Lactams

**Figure 1:**
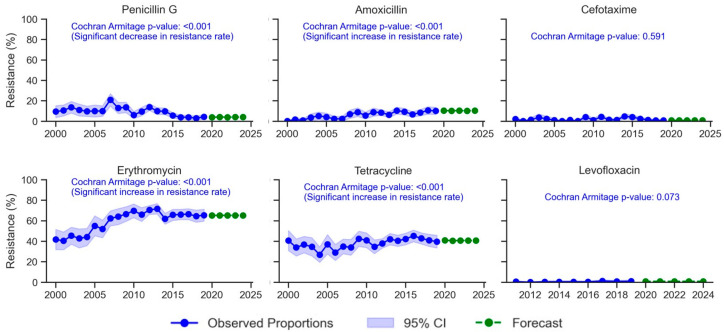
Evolution of resistance rates to penicillin G, amoxicillin, cefotaxime, erythromycin, tetracycline, and levofloxacin among *S. pneumoniae* isolates in Tunisia from 2000 to 2019. CI: confidence interval.

Figure 1 depicts the evolution of *S. pneumoniae* resistance over two decades, 2000–2019, and the forecasted resistance rates post-2019. The resistance rate to penicillin G significantly decreased from 9–13.7% during 2000–2002 to 2.9–4.3% in 2016–2019 (*p* < 0.001). However, this rate increased temporarily in specific years such as 2007 (20.9%) and 2012 (13.8%). Using time series analysis, the prevalence of resistant strains to penicillin G was predicted to remain below 10% during 2020–2024.

A steady significant increase in amoxicillin resistance patterns was observed from 0% during 2000 to 10% in 2019 (*p* < 0.001). The resistance to amoxicillin was estimated to be around 10–11% in the subsequent years.

Cefotaxime resistance remained low throughout the study period, with the highest proportion of resistant strains recorded in 2014 (4.7%). The prevalence of cefotaxime resistance was estimated to be around 1% during 2020–2024.

#### 2.2.2. Resistance Trends to Other Antibiotics

Erythromycin resistance significantly increased over the study period (*p* < 0.001), rising from 41.7% in 2000 to a peak of 71.6% in 2013 before stabilizing between 61.9% and 66.1% during 2014–2019. Projections for 2020–2024 estimated that the erythromycin resistance rate would remain around 65%.

Tetracycline resistance decreased from 40.6% in 2000 to 34% in 2008, with a notable decline to 26.6% in 2004. Between 2009 and 2019, resistance rates fluctuated between 34.6% and 45.2%. For the period 2020–2024, tetracycline resistance was projected to be around 40%.

Levofloxacin resistance remained below 0.6% from 2011 to 2016, then increased to 0.7–1.2% between 2017 and 2019. This value was projected to persist at approximately 1% from 2020 to 2024.

### 2.3. Evolution of S. pneumoniae Antimicrobial Resistance by Infection Site and Age

**Figure 2:**
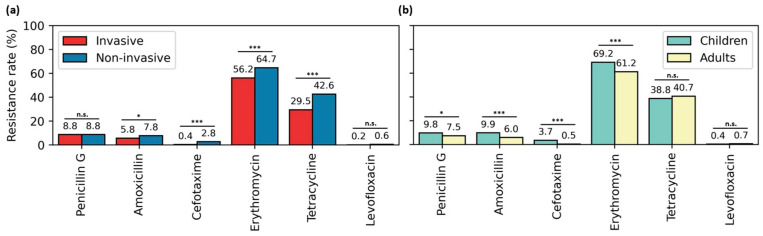
Antimicrobial resistance rates of pneumococcal isolates by site of infection (**a**) and age (**b**) in Tunisia (2000–2019). *p*-values are indicated as follows: * 0.01 < *p* ≤ 0.05; *** *p* ≤ 0.001; n.s.: not significant.

**Figure 3:**
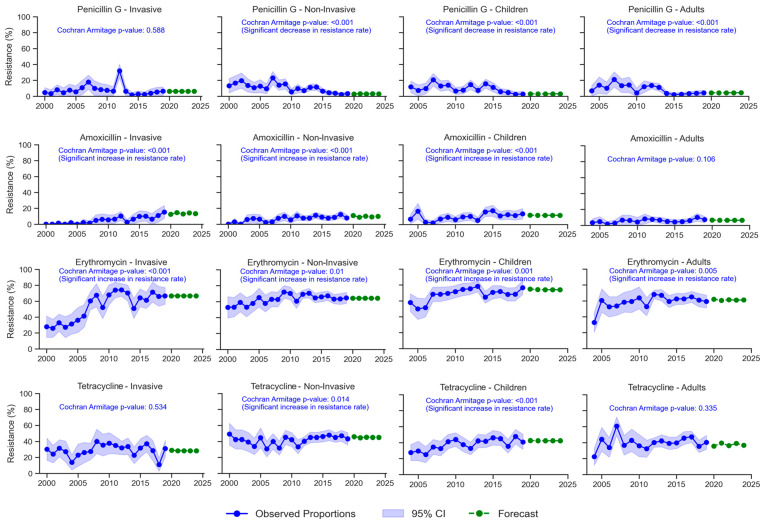
Trends of *S. pneumoniae* resistance to penicillin G, amoxicillin, cefotaxime, erythromycin, tetracycline, and levofloxacin according to infection site (invasive and non-invasive) and age range (adults and children) in Tunisia from 2000 to 2019. CI: confidence interval.

The overall resistance rates to selected antibiotics according to infection site (invasive and non-invasive) and age (adults and children) are displayed in Figure 2, and the corresponding resistance trends are illustrated in Figure 3.

#### 2.3.1. Resistance Rates to β-Lactams by Infection Site and Age

The overall rate of penicillin G resistance was 8.8% in both invasive and non-invasive sites. This rate was slightly higher in children (9.8%) than adults (7.5%). A significant decline in resistance trends for non-invasive infections and for both age groups was noted, while no notable trend was detected for invasive infections.

For amoxicillin, non-invasive strains exhibited a higher resistance rate than invasive strains (7.8% vs. 5.8%; *p* = 0.024). Pediatric isolates were 1.65 times more resistant to amoxicillin than those from adults (9.9% vs. 6%; *p* < 0.001). A substantial increase in amoxicillin resistance was observed across invasive and non-invasive sites and among pediatric samples.

The overall cefotaxime resistance was 0.4% in invasive samples and 2.8% in non-invasive samples. Resistance was significantly higher in children than in adults (3.7% vs. 0.5%, *p* < 0.001). However, no statistically significant trend in cefotaxime resistance was found across any category.

#### 2.3.2. Resistance Rates to Other Antimicrobials by Infection Site and Age

Erythromycin resistance was 56.2% in invasive strains compared to 64.7% in non-invasive strains (*p* < 0.001). The resistance rate was significantly higher among pediatric isolates than adults (69.2% vs. 61.2%; *p* < 0.001). A statistically significant increase in erythromycin resistance was observed across invasive and non-invasive samples and in both age groups.

The overall resistance rate to tetracycline was statistically lower in invasive strains than in non-invasive strains (29.5% vs. 42.6%; *p* < 0.001). Adult strains showed a slightly higher resistance rate (40.7%) than pediatric strains (38.8%). A significant increase in tetracycline resistance was detected among non-invasive sites and for pediatric isolates.

Levofloxacin resistance was only detected in 0.2% of invasive strains and 0.6% of non-invasive strains. The resistance rate in pediatric isolates (0.4%) was lower than in adults (0.7%). No statistically significant change in the levofloxacin resistance trend was observed in any category.

### 2.4. Distribution of S. pneumoniae Serotypes and Their Associated Resistance Rates

A total of 903 serotyped isolates were collected from the 2 centers (Habib Bourguiba University Hospital of Sfax and Children’s Hospital of Tunis), with 700 (77.5%) belonging to the top 8 serotypes. These eight most common serotypes are, in descending order: 14 (n = 203), 19F (n = 158), 19A (n = 81), 23F (n = 80), 3 (n = 60), 6B (n = 53), 6A (n = 35), and 9V (n = 30). Five (6B, 9V, 14, 19F, 23F; n = 524) out of the eight most common serotypes identified are covered by PCV-7 and PCV-10, providing a combined coverage rate of 74.9%, whereas all eight predominant serotypes are included in PCV-13. These serotypes remained prevalent between 2012 and 2018, with a slight downward trend for serotypes 14 and 19F, which led to a small decrease in the vaccine coverage of PCV-7, PCV-10, and PCV-13 (Figure S1).

The resistance rates of the most dominant *S. pneumoniae* serotypes to various antibiotics are provided in Table 1. Among the top eight most prevalent serotypes in this study, serotypes 14 and 9V exhibited the highest resistance to amoxicillin, ranging from 65.5% to 71.9%. These serotypes and 23F also showed significant resistance to penicillin G (24.7%–27.7%) and cefotaxime (3.4–6.8%). Most serotypes demonstrated high resistance to erythromycin, exceeding 75%, except for serotypes 3 (20%) and 9V (50%). The most resistant serotypes to tetracycline were serotypes 19F, 19A, and 6B. All serotypes remained largely susceptible to levofloxacin.

**Figure 4:**
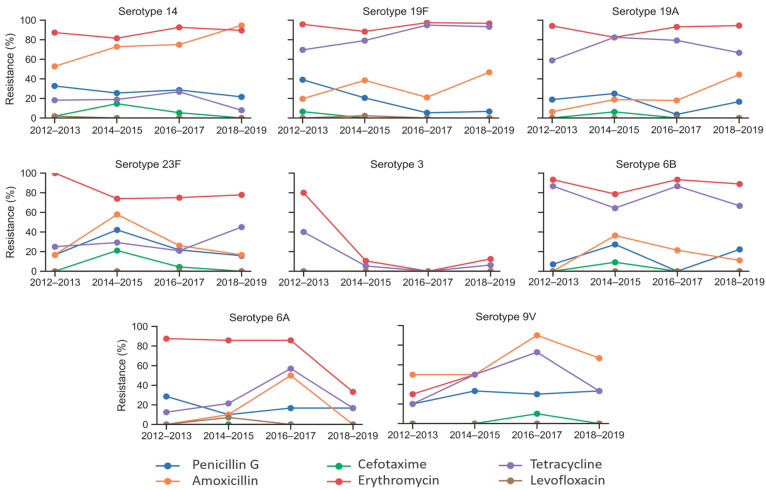
Trends of antimicrobial resistance within eight predominant *S. pneumoniae* serotypes (14, 19F, 19A, 23F, 3, 6B, 6A, and 9V) in Tunisia between 2012 and 2019.

Resistance trends varied among the top eight serotypes, as shown in Figure 4. Serotype 3 exhibited a sharp decline in resistance to erythromycin and tetracycline, which decreased from 80% and 40% in 2012–2013 to 12.5% and 6.2% in 2018–2019, respectively. Other serotypes demonstrated fluctuating resistance patterns throughout the study period. Nevertheless, notable decreases were observed in erythromycin resistance for serotypes 23F and 6A, which fell from 100% and 88% in 2012–2013 to 78% and 33% in 2018–2019, respectively. Penicillin G resistance also declined for serotypes 19F and 6A, decreasing from 39% and 29% in 2012–2013 to 7% and 17% during 2018–2019, respectively. Conversely, amoxicillin resistance increased among serotypes 14, 19F, 19A, and 9V over the study period.

## 3. Discussion

Large national surveillance studies monitoring the trends in *S. pneumoniae* resistance patterns and the circulating serotypes are vital for understanding the local epidemiology of pneumococcal disease. This multicenter study evaluated for the first time the prevalence, trends, and antimicrobial resistance patterns of *S. pneumoniae* in Tunisia over 20 years (2000–2019). It also analyzed the antibiotic resistance among the most common serotypes of *S. pneumoniae* from 2012 to 2019 in Tunisia.

Among 4284 *S. pneumoniae* isolates, the overall penicillin G resistance rate was estimated at 8.8%. Over 20 years, a statistically significant downward trend was observed in *S. pneumoniae* resistance to penicillin G. Zhang et al. evaluated 3929 *S. pneumoniae* isolates obtained from 150 centers globally as part of the Tigecycline Evaluation and Surveillance Trial (TEST) between 2015 and 2017, and reported a penicillin resistance rate of 13.6% [14]. A study from Jordan reported a 10.3% (n = 544) rate of non-susceptibility to penicillin during 2000–2018 [2]. Conversely, China (1.3% in non-invasive isolates, n = 6132) and Spain (2% in IPD isolates, n = 7133) exhibited lower rates of penicillin G resistance [15,16]. In contrast, increased resistance rates to penicillin G were observed in non-invasive *S. pneumoniae* isolates obtained from children in the US (42.4%, n = 7605) [9]. From 2010 to 2021, there was no significant change in the trend of penicillin G resistance at the non-meningitis breakpoint in the UAE, which varied from 0% to 5% [1]. Treatment with high-dose penicillin G was effective in 87.1% of pneumonia cases in the study conducted by Komagamine et al. [17]. This therapeutic strategy may be empirically useful in treating pneumococcal pneumonia in countries with a low resistance rate to penicillin G, such as Tunisia.

This study revealed a statistically significant upward trend in *S. pneumoniae* resistance to amoxicillin across invasive, non-invasive, and pediatric isolates. In contrast, a downward trajectory in amoxicillin resistance was observed in the UAE from 2010 to 2021 [1]. In this study, the proportion of amoxicillin resistance was around 7%. These findings were lower compared to rates observed in Morocco (21.4%, n = 645) [18].

Throughout the study period, *S. pneumoniae* isolates demonstrated high susceptibility to cefotaxime, with a resistance rate ranging from 0.4% to 3.7% across all categories, and no significant resistance trend was identified. Resistance rates were comparable among isolates obtained from Tunisia, the UAE, and Malaysia [1,13,19]. In contrast, increased resistance to extended-spectrum cephalosporins was seen in invasive isolates obtained from the US, Jordan, and Taiwan [2,3,9]. Third-generation cephalosporins are the preferred treatment for IPD; however, rising resistance among invasive isolates should be considered before initiating treatment.

Over the study duration, we observed a rising trend in *S. pneumoniae* resistance to erythromycin across all categories, with resistance rates markedly higher than for β-lactams, ranging from 40.3% to 71.6%. These results correlate with three Tunisian studies [4,12,13] and align with the global pattern of erythromycin resistance observed in *S. pneumoniae* [1,2,9,15,19,20,21,22,23]. Given their broad-spectrum activity, macrolides are widely prescribed antibiotics for treating CAP. However, their increased use has resulted in elevated macrolide resistance, emphasizing the requirement for targeted stewardship programs to tackle resistance in *S. pneumoniae* isolates.

In this report, non-invasive *S. pneumoniae* isolates displayed increased resistance to tetracycline compared to invasive isolates (42.6% vs. 29.5%). The findings align with Ben Ayed et al.’s study, which demonstrated significantly higher non-susceptibility to tetracycline among non-invasive isolates than invasive isolates (47.2% vs. 36.8%) [13]. This study illustrated that levofloxacin exhibited the highest activity against *S. pneumoniae* isolates, and no significant resistance patterns were observed over the 20 years in Tunisia. Similar susceptibility patterns for tetracycline and levofloxacin were observed globally [1,13,19,21,22,24,25]. A large surveillance study conducted across 400 sites from 1997 to 2016 assessed the activity of antimicrobial agents against 65,993 pneumococcal isolates sourced from Latin America, North America, Europe, and the Asia–Pacific region [26]. A decreasing trend in tetracycline sensitivity over the first 12–14 years was observed in this report, which then increased in the last 6–8 years [26]. In the same study, levofloxacin was found to be the second most sensitive agent behind linezolid [26]. However, tetracycline resistance in *S. pneumoniae* isolates from Morocco showed a downward trend with a total resistance rate of 20.9% [18], while in China, isolates obtained from pediatric samples showed a markedly high rate of resistance to tetracycline (93.6%) [15]. In contrast with our study and other global studies, Jordan displayed a higher prevalence of non-susceptibility (23.5%) to levofloxacin, with a statistically significant increase from 0% in 2000 to 26.8% in 2018. The authors attribute the rising prevalence to the growing use of levofloxacin in treating pneumococcal disease in Jordan [2].

Differences in the prevalence of pneumococcal serotypes across geographical locations can be attributed to variations in age demographics, vaccine coverage rates, immunization efforts, and AMR patterns [6]. Serotypes 1 and 3 are frequently seen in pneumonia cases, whereas serotypes/serogroups 6, 10, and 23 are predominantly identified in patients with meningitis. Globally, serotypes 14, 19F, and 19A are most frequently associated with cases of IPD [27]. In this study, serotypes 14, 19F, 19A, 23F, 3, 6B, 6A, and 9V emerged as the predominant serotypes, representing 77.5% of the total serotypes (n = 903). These results align with global epidemiological patterns [6,27] and are in concordance with three previous studies conducted in Tunisia [4,12,13]. Five (6B, 9V, 14, 19F, 23F; n = 524) out of the eight most common serotypes identified in this study are covered by PCV-7 and PCV-10, providing a combined coverage rate of 74.9%, whereas all these eight predominant serotypes are included in PCV-13.

In Tunisia, the prevalence of the main serotypes remained mostly stable, due to the limited use of PCVs in the country before 2019. In April 2019, PCV-10 was integrated into the national immunization program, which may lead to significant changes in the serotype distribution of *S. pneumoniae*. As the proportion of vaccine-covered serotypes is high, this immunization will be beneficial in preventing vaccine-type CAP and IPD in Tunisia.

The resistance of *S. pneumoniae* to antibiotics varies among different serotypes. In the present study, serotypes 14 (27.7%), 9V (27.6%), 23F (24.7%), 19F (19.6%), and 6A (17.2%) were identified as the most penicillin G-resistant serotypes. Serotype 14 displayed the highest resistance rate against amoxicillin (71.9%), followed by 9V (65.5%). Apart from serotype 3, the seven remaining serotypes displayed high resistances to erythromycin, while all these eight serotypes exhibited a high susceptibility to cefotaxime and levofloxacin. In Spain, serotypes 11A, 19A, 14, and 9V [16]; in China, 19F, 19A, 6A, 6B, 14, and 23F [28]; and in European countries, 14, 19A, and 15A [29] showed higher resistance rates to penicillin. Before the implementation of PCVs, serotypes 6B, 9V, 14, 19F, and 23F were predominantly linked to elevated resistance to β-lactams [13]. In Tunisia, serotypes 6A and 6B [12] and in the Philippines, serotypes 1, 6, 23, and 24 [30] were linked to a high level of erythromycin resistance. Hackel et al. conducted an analysis of 2173 IPD isolates from a global database to assess the relationship between serotypes and antibiotic susceptibilities [31]. Their study revealed that serotypes 19A, 19F, 35B, 6A, 6B, 23A, 9V, 15A, and 14 showed increased levels of penicillin resistance [31]. Additionally, they found that serotypes 19A, 6A, 19F, 6B, 15A, 9V, and 14 were associated with higher rates of erythromycin resistance [31]. According to another Tunisian study, serotypes 19A, 19F, 23F, and 6B were associated with MDR in *S. pneumoniae* isolates [13]. In Greece, serotypes 15A,17F,19A, and 19F [32]; in China, 6A, 23F, 6B, 19F, and 15B [33]; in Korea, 23A, 11A, 19A, and 15B [20]; and in European countries, 15A, 19A, and 14 [29] exhibited the highest levels of MDR. In the present study, serotypes 23F, 14, 9V, and 6B exhibited higher rates of MDR.

This study has some limitations that should be acknowledged. The isolates were obtained only from university hospital centers. The serotyping data were limited to two hospitals (Habib Bourguiba University Hospital of Sfax and Children’s Hospital of Tunis) and to the 2012–2019 period, which may not be representative of the national distribution of pneumococcal serotypes. These two hospitals were the main providers of pneumococcal strains in this study. This study did not include the molecular analysis of the *S. pneumoniae* isolates, which could reveal more information about the genetic diversity of the strains circulating in Tunisia and their underlying mechanisms of resistance.

## 4. Materials and Methods

### 4.1. Study Sites and Data Collection

This retrospective antimicrobial resistance surveillance study gathered data from 2000 to 2019 from eight participating university hospital centers located in different regions of Tunisia. Between 2000 and 2010, antimicrobial resistance surveillance was conducted at four centers (Habib Bourguiba University Hospital of Sfax, Children’s Hospital of Tunis, Charles Nicolle Hospital of Tunis, and Bone Marrow Transplant Center of Tunis). From 2011, four additional centers (La Rabta Hospital of Tunis, Kassab Institute of Orthopaedics of Tunis, Aziza Othmana Hospital of Tunis, and Abderrahmen Mami Hospital of Tunis) were included in the surveillance, increasing the total number of participating centers to eight. The study was approved by the Medical School of Sfax ethics committee (ethics committee decision N 12/23). Isolates were identified using conventional procedures, including Gram staining, optochin sensitivity, and bile solubility tests. For this analysis, the clinical isolates obtained from sterile body sites including blood culture and cerebrospinal fluid (CSF) were defined as invasive isolates, while those from upper and lower respiratory tract sites were classified as non-invasive. Data regarding the annual number of *S. pneumoniae* strains, the proportion of invasive versus non-invasive strains, and the percentage of AMR were collected. Patients under 16 years of age were categorized as pediatric, while those 16 years and older were classified as adults. The analysis did not include duplicate strains from the same patient.

### 4.2. Antimicrobial Susceptibility Testing and Serotyping

Antimicrobial susceptibility testing (AST) was conducted following the European Committee on Antimicrobial Susceptibility Testing (EUCAST) guidelines that were applicable at the time of sample collection [34]. All clinical isolates were tested using the disk diffusion method against the following antimicrobial agents: penicillin G, amoxicillin, cefotaxime, erythromycin, tetracycline, and levofloxacin. Minimum inhibitory concentrations (MICs) for penicillin G, amoxicillin, cefotaxime, and levofloxacin were determined using the E-test method following the manufacturer’s instructions (bioMérieux) for all strains. An internal quality control test was conducted by testing the control strain *S. pneumoniae* ATCC49619.

Data on pneumococcal serotypes were gathered from two hospitals, Habib Bourguiba University Hospital of Sfax and Children’s Hospital of Tunis, from 2012 to 2019. Multiplex PCR assays that covered 29 serotypes were used to identify the pneumococcal serotypes, following the guidelines of the World Health Organization (WHO) [35] and the Pai et al. method [36]. These serotypes were selected based on their regional prevalence, as reported previously [13]. The latex agglutination technique was employed for cases where the serotype was not identified with PCR.

### 4.3. Time Series Analysis

Over two decades, from 2000 to 2019, a time series analysis was conducted to track the evolution of *S. pneumoniae* resistance in Tunisia. We also evaluated the prevalence of AMR by stratifying strains into invasive and non-invasive categories and by age groups. Moreover, the association between serotypes and antibiotic resistance was also investigated from 2012 to 2019 in two Tunisian hospitals (Habib Bourguiba University Hospital of Sfax and Children’s Hospital of Tunis).

### 4.4. Statistical Analysis

The evolution of *S. pneumoniae* resistance over time was represented by line graphs, categorizing strains into invasive and non-invasive types and by age groups. An autoregressive integrated moving average (ARIMA) model was used to predict resistance trends. We conducted the Cochrane Armitage test for trend to determine the statistical significance of the *S. pneumoniae* resistance trends over the 2000–2019 period. The chi-square test was employed to compare resistance rates according to the infection site and the patient’s age. Line graphs were used to illustrate the resistance patterns of the eight top serotypes to various antibiotics. A *p*-value below 0.05 was defined as statistically significant.

## 5. Conclusions

In conclusion, this study demonstrated a statistically significant upward trend in *S. pneumoniae* resistance to amoxicillin, erythromycin, and tetracycline from 2000 to 2019 in Tunisia. Additionally, before the implementation of PCV-10 in the national immunization program, the most prevalent *S. pneumoniae* serotypes in decreasing order were 14, 19F, 19A, 23F, 3, 6B, 6A, and 9V. Serotypes 23F, 14, 9V, and 6B displayed the highest levels of multi-drug resistance, whereas serotype 3 showed susceptibility to all tested antibiotics. The pneumococci showed the strongest resistance to erythromycin and tetracycline, making them unsuitable for treating pneumococcal infection in Tunisia. However, penicillin G, cefotaxime, and levofloxacin can be used empirically to treat infectious diseases caused by *S. pneumoniae* in Tunisia. Implementing antimicrobial stewardship programs that prioritize the rational use of antibiotics, effective pneumococcal vaccination initiatives, and continued surveillance of prevalent serotypes will be essential strategies for reducing the overall disease burden associated with *S. pneumoniae* infections in Tunisia.

## Figures and Tables

**Table 1:** The distribution of the main eight *S. pneumoniae* serotypes (n = 700 isolates) and their rate of antimicrobial resistance.

Serotype	Penicillin	Amoxicillin	Cefotaxime	Erythromycin	Tetracycline	Levofloxacin	MDR ^1^
14	54 (27.7%)	141 (71.9%)	11 (5.6%)	176 (87.6%)	38 (18.8%)	1 (0.5%)	47.3%
19F	30 (19.6%)	46 (30.1%)	3 (2%)	149 (94.3%)	131 (82.9%)	1 (0.6%)	35.4%
19A	11 (14.1%)	17 (21.8%)	1 (1.3%)	74 (91.4%)	59 (72.8%)	0	38.3%
23F	18 (24.7%)	22 (30.6%)	5 (6.8%)	61 (79.2%)	24 (30%)	0	53.8%
3	0	0	0	12 (20%)	6 (10.2%)	0	0%
6B	6 (12.5%)	8 (16.7%)	1 (2.1%)	47 (88.7%)	41 (77.4%)	0	45.3%
6A	5 (17.2%)	4 (13.8%)	0	27 (77.1%)	9 (25.7%)	1 (2.9%)	17.1%
9V	8 (27.6%)	19 (65.5%)	1 (3.4%)	15 (50%)	14 (46.7%)	0	46.7%

^1^ MDR: multi-drug resistance.

## Data Availability

The original contributions presented in the study are included in the article. Further inquiries can be directed to the corresponding author.

## Supplementary Materials

The following supporting information can be downloaded at: https://www.mdpi.com/article/10.3390/antibiotics14020171/s1, Figure S1. Evolution of serotype distribution (a) and pneumococcal vaccine coverage (b) over the study period.

## Abbreviations

The following abbreviations are used in this manuscript:

AMR	Antimicrobial drug resistance
ARIMA	Autoregressive integrated moving average
AST	Antimicrobial susceptibility testing
CAP	Community-acquired pneumonia
CDC	Center for Disease Control and Prevention
CSF	Cerebrospinal fluid
EUCAST	European Committee on Antimicrobial Susceptibility Testing
IPD	Invasive pneumococcal disease
MDR	Multi-drug resistance
MIC	Minimum inhibitory concentration
PCR	Polymerase Chain Reaction
PCV	Pneumococcal conjugate vaccine
WHO	World Health Organization
