# Supplementary material for: The Evolution of the Antimicrobial Resistance of Streptococcus pneumoniae in Tunisia: A Multicentric Analysis over Two Decades (2000–2019)
Source: Antibiotics (Basel). 2025 Feb 10;14(2):171. doi: 10.3390/antibiotics14020171 (PMC11851641; doi:10.3390/antibiotics14020171)
Supplement: Supplementary file 1 [file antibiotics-14-00171-s001.zip › antibiotics-3442121-supplementary.pdf]

(a)

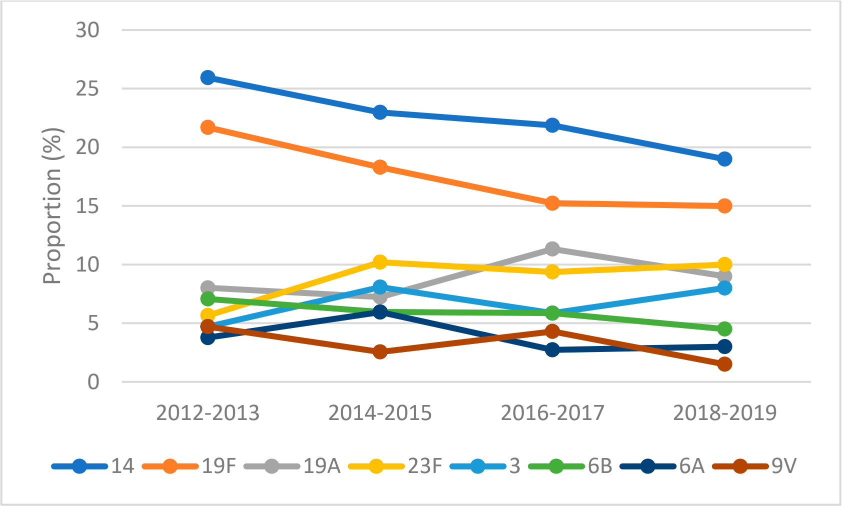

(b)

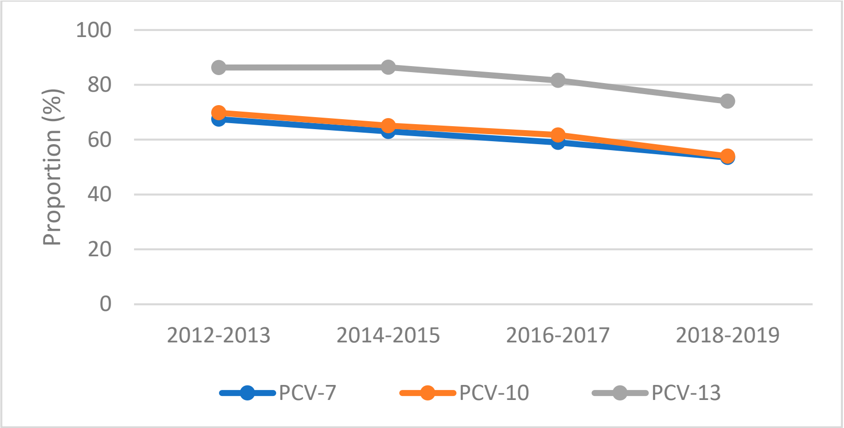

Figure S1. Evolution of serotype distribution (a) and pneumococcal vaccine coverage (b) over the study period
